# High Pressure Processing and its Application to the Challenge of Virus-Contaminated Foods

**DOI:** 10.1007/s12560-012-9094-9

**Published:** 2012-11-20

**Authors:** David H. Kingsley

**Affiliations:** USDA Agricultural Research Service, Delaware State University, Dover, DE 19901 USA

**Keywords:** High pressure processing, Foodborne viruses, Bivalve shelfish, Produce, Oysters, Clams, Mussels

## Abstract

High pressure processing (HPP) is an increasingly popular non-thermal food processing technology. Study of HPP’s potential to inactivate foodborne viruses has defined general pressure levels required to inactivate hepatitis A virus, norovirus surrogates, and human norovirus itself within foods such as shellfish and produce. The sensitivity of a number of different picornaviruses to HPP is variable. Experiments suggest that HPP inactivates viruses via denaturation of capsid proteins which render the virus incapable of binding to its receptor on the surface of its host cell. Beyond the primary consideration of treatment pressure level, the effects of extending treatment times, temperature of initial pressure application, and matrix composition have been identified as critical parameters for designing HPP inactivation strategies. Research described here can serve as a preliminary guide to whether a current commercial process could be effective against HuNoV or HAV.

Although there are a myriad of enteric viruses that can be transmitted orally, at present the two principal foodborne threats are human norovirus (HuNoV) and hepatitis A virus (HAV). Inactivation of HAV and HuNoV is very challenging because these viruses are environmentally stable, and able to persist in cool, damp, and dark environments for periods of months, or even a year or more. They are resistant to low pH, detergents, and organic solvents, and are more resistant than bacteria to water treatments, such as chlorination. HAV and HuNoV are generally inactivated by cooking but due to technical difficulties associated with laboratory propagation of these viruses, validation of thermal inactivation conditions within specific foods is generally lacking.

High pressure processing (HPP) has emerged as a novel technology for food processing where foods can maintain their raw character and flavor. Applications of HPP include its use as a “cold pasteurization” method for fruit juices, a means of sanitizing packaged ready-to-eat meats, and inactivation of spoilage enzymes to enhance refrigeration shelf-life of avocados and guacamole. High pressure can also separate raw shellfish meat from its shell. This has been done successfully for lobsters and crabs, as well as for bivalve shellfish such as oysters and clams. In addition to facilitating commercial shucking of oysters, this technology is also used as an intervention to inactivate *Vibrio vulnificus* bacteria found in oysters grown in warm waters. High pressure is viewed favorably by regulatory agencies since food treatment simply involves exposing foods to high pressure. HPP is not without its limitations however. It is generally ineffective against bacterial spores (Akhtar et al. 2009; Shearer et al. 2000) and commercial HPP equipment is expensive. As a result, its application is generally limited to refrigerated foods and for use by high throughput commercial operations.

For the last decade, the U.S. Dept. of Agriculture, Agriculture Research Service Laboratory at Delaware State University in conjunction with its collaborators have endeavored to evaluate the utility of high pressure processing as a mitigation strategy for foodborne viruses. Before this work, information about the potential of foodborne viruses to be inactivated by high pressure was virtually non-existent.

## High Risk Foods

Beyond contamination at the point of service through non-hygienic kitchen or server practices, two food types present elevated virus transmission risk due to the potential for contamination during production or harvest. The first are fruits and vegetables that are often hand-picked, providing the potential for fecally contaminated fingers to contact the produce (Baert et al. 2011). Furthermore, irrigation of produce with non-potable water that has been subjected to human fecal contamination is another potential source of virus contamination (Hall et al. 2012). In fact, there is some suggestion that viruses may actually sequester themselves within produce when irrigated by non-potable water, rather than just contaminate surfaces (Chancellor et al. 2006; Urbanucci et al. 2009; Wei et al. 2011). Produce and their products are often imported from developing countries with less stringent hygienic standards where labor and production costs are low. In some cases, wash and toilet facilities at harvest locations may not even exist. Notable outbreaks of hepatitis A have been associated with green onions imported into the US that were used to make salsa served in a Mexican-style restaurant chain (Anon 2003) and with frozen strawberries served in school lunch programs (Niu et al. 1992). For norovirus, there have recently been a number of outbreaks associated with raspberries (Sarvikivi et al. 2012).

The second food type presenting elevated virus risks are bivalve shellfish, such as oysters, clams, cockles, and mussels. Shellfish are filter feeders that readily bioconcentrate virus pathogens from the water column, filtering as much as 250 liters/day/oyster (Loonsanoff 1958) and as a result, may concentrate viruses as much as 1,000-fold (Canzonier 1971) from the surrounding water. As mentioned previously, cooking is generally thought sufficient to inactivate viruses within shellfish, but validation is lacking, and many consumers eat oysters and clams either raw, or only lightly-cooked. While mussels are more commonly cooked, these bivalves are also consumed raw in some regions such as the Mediterranean. Currently, there is no effective strategy to eliminate pathogenic human viruses from shellfish. Depuration, a process in which shellfishes are held in tanks of clean water and allowed to pump for a few days is a relatively effective means of reducing pathogenic bacteria of fecal origin; however, fecal viruses are not effectively eliminated (Grohmann et al. 1981; Love et al. 2010). In fact, characterizing the ability of virus to persist in oysters, our laboratory demonstrated that HAV could be detected 6 weeks after the contamination of live pumping shellfish held under simulated depuration conditions (Kingsley and Richards 2003). Our laboratory has also shown that these viruses can sequester themselves within hemocyte cells inside the oyster tissues (Provost et al. 2011).

## The Norovirus Problem

HuNoVs cause the majority of US foodborne illness and are thought responsible for 11 and 25 % of foodborne deaths and hospitalizations, respectively (Scallan et al. 2011). While often quite unpleasant for healthy individuals, norovirus infection is normally self-limiting, resulting in 24–48 h of diarrhea and vomiting. Complications can occur as a result of dehydration, and in the rare case of patients undergoing stem cell transplants, this common virus can become lethal (Schwartz et al. 2011). Currently, the frequency of HuNoV infection in the US is approximately 10–15 % per person per annum (Scallan et al. 2011). HuNoV is now so prevalent that untreated sewage from virtually any common population source should be considered to contain viable norovirus. HuNoVs have not been reproducibly replicated in vitro and currently there are no practical animal models, presenting a significant problem for norovirus research and control efforts. Thus, research has focused on propagable, genetically related surrogates such as feline calicivirus (FCV; Buckow et al. 2008) and murine norovirus (MNV; Wobus et al. 2006). Other less commonly used surrogates include San Miguel sea lion virus (Burkhardt et al. 2002), canine calicivirus (Urbanucci et al. 2009), and the recently discovered Tulane virus (Farkas et al. 2008), which is a primate norovirus. Direct assessment of HuNoV infectivity is currently only feasible using expensive and logistically complicated human volunteer trials. There are two sources of infectious HuNoV which result in exposure and illness. The first is by fecal contamination of food or water from a HuNoV-infected person. The second less-appreciated exposure route is a norovirus vomiting event, which results in aerosolized norovirus particles exposing persons in the general vicinity and coating environmental and food preparation surfaces (Marks et al. 2003; Friesema et al. 2009). In commercial kitchens and high population density institutional settings, this latter exposure route is a substantial issue.

## The Hepatitis A Problem

With the advent of vaccinations, HAV is becoming increasingly rare in the developed world (Jacobsen and Koopman 2004), while it remains endemic in the developing world. There is an age-associated virulence with this virus. If acquired by older children and adults, hepatitis A virus can cause a medically serious illness characterized by jaundice 30–60 days post-exposure (Franco et al. 2012). For immunologically naïve persons over the age of 50, there is a 1 % mortality associated with HAV infection (Fitzsimons et al. 2010). Single exposure to HAV generally results in long-term, if not life-long, immunity to the virus. For children exposed at an early age, the infection is often unapparent, not resulting in serious illness, but providing long-term resistance to HAV. As a result, developing countries may be less inclined to focus limited public health efforts on a virus for which the country’s population is largely immune. With increasing international trade, this situation presents a serious threat to developed nations where large population segments are susceptible to HAV. While HAV has been adapted to tissue culture, wild-type strains are extremely difficult to propagate in vitro (Lemon et al. 1991), making routine bioassay impractical. Thus, HAV research is typically performed using a tissue culture-adapted HM-175 HAV strain (Cromeans et al. 1987).

## Other Foodborne Viruses

HAV and HuNoV are members of the *Picornaviridae* and the *Caliciviridae* families, respectively. A number of different picornaviruses (enterovirus genera) also present some foodborne concern since they are commonly found in human stool and have been associated with various chronic syndromes (Riecansky et al. 1989; Berger et al. 2000; Roivainen et al. 2002; Yin et al. 2002). These include the echo- and parecho- and the coxsackie-viruses. Although not known to be associated with chronic syndromes, Aichivirus—another picornavirus—has been documented with shellfish-borne gastroenteritis outbreaks in both Asia and Europe (Le Guyader et al. 2008; Yamashita et al. 1998). Among the *Caliciviridae*, the sapoviruses are much less common than norovirus, but have also been associated with foods such as oysters (Lizuka et al. 2010; Ueki et al. 2010). Originally classified as a calicivirus, but now classified in its own genera based on some distinct molecular biologic differences, hepatitis E virus (HEV) may be an emerging virus of some concern. There are four genotypes of this virus with types 1 and 2 being associated with medically serious person-to-person (fecal–oral) route transmission in underdeveloped nations. The principal mode of HEV transmission is thought to be water, but association with foods such as undercooked pork, game meat, and oysters have been documented (Meng 2011; Nelson et al. 2011). Genotypes 1 and 2 are endemic to the Asian subcontinent and Africa, but are considered exotic in North America and Europe. Genotypes 3 and 4 appear to be commonly associated with swine and wild game animals worldwide and, as a result, are considered zoonotic (Meng 2011). However, the routine presence of zoonotic HEV in uncooked pork livers sold in US markets has been demonstrated (Feagins et al. 2007). Reports of infection and illness with zoonotic HEV are rare, but, when reported, are typically associated with raw meat consumption and persons who have underlying health issues. Other viruses that spread by fecal-oral route and may have foodborne transmission potential include the rotaviruses, adenoviruses, and the astroviruses.

## Characteristics of HPP

Typical pressures used for commercial food processing machines are as high as 600 Megapascals (MPa). As a unit of measuring pressure, 1 MPa equals 9.87 atmospheres or 145 pounds per square inch. Commercial HPP units are often quite large with capacities exceeding several hundred liters. Processing is by the batch with machines filled, treated for short intervals (usually less than 5-min), and then emptied. Commercial units are almost exclusively water based, but research units can use water, oil, or alcohol as the pressure application medium. Although HPP is classified as a non-thermal process, an adiabatic heating effect occurs under pressure that can be substantial with increasingly greater adiabatic heating effects observed for water, oil, and alcohol pressure medium (Balasubramanian and Balasubramaniam 2003). Thus, while the temperature before pressure application of 600-MPa may be at 25 °C, the expected temperature achieved under pressure assuming a 3.5 °C adiabatic heating per 100-MPa for a water-based unit would increase to approximately 46 °C. For oil- and alcohol-based units, adiabatic heating is proportionally greater with increasing pressure. It is important to note that even under pressure, temperatures above 60 °C may have some thermal pathogen inactivation effects in and of themselves. For smaller units, adiabatic heat will typically dissipate through the vessel walls and re-equilibrate toward the outside temperature surrounding the vessel. However, larger commercial scale units are more prone to retain adiabatic heat due to larger vessel volume to surface ratios. Time to achieve pressure, commonly termed “come-up” time, is variable with different units and probably does contribute to the inactivation observed. For most machines, pressure release is achieved in a few seconds, if not almost instantaneously. It is also important to recognize that when pressure is achieved, that pressure is experienced by the whole sample for the entire period. This contrasts with thermal cooking methods where time is required to achieve the appropriate internal temperature for a food item.

## Oysters and Commercial HPP

For shellfish, HPP is currently used as an established and well-accepted intervention for *Vibrio vulnificus* (Vv) in the US (Iwamoto et al. 2010). Research also suggests that HPP can be used to control *Vibrio parahemolyticus*, another endemic bacterium that can cause gastroenteritis (Kural et al. 2008). HPP has an additional labor-saving benefit since pressure completely separates the meat from the shell, greatly facilitating the shucking process and improving the presentation quality of on-the-half-shell shellfish. Reportedly, HPP treatment of shellfish also can extend the refrigerated shelf-life of oysters via reduction of spoilage bacteria (He et al. 2002). The current pressure used to treat commercial shellfish is 275–300-MPa for several minutes, but oysters are reported to still taste good when treated as high as 400 MPa (Lopez-Caballero et al. 2000; Cruz-Romero et al. 2004).

## Pressure Sensitivity of HAV

Initially, to evaluate HPP effectiveness against the tissue culture-adapted HM-175 HAV strain, experiments were performed using a custom-built oil-based unit with pressures applied at room temperature. Results indicated that pressure had no effect on HAV stock in DMEM tissue culture media until pressures of above 300 MPa were applied. Complete inactivation of a 7-log_10_ HAV stock was observed at 460 MPa (Kingsley et al. 2002). Preliminary experiments to determine how seawater and extended time of pressure application might affect inactivation were performed (Kingsley et al. 2002). HAV stock was mixed with 9 parts seawater and 5-min treatments were extended to 15 min. Results indicated that extending the pressure treatment did enhance inactivation, but the amount of pressure applied had more influence on the amount of inactivation than extending the time of pressure application. Seawater, which elevated the sample salinity to approximately 2.5 %, was observed to reduce the effectiveness of high pressure inactivation. Grove et al. (2008) also subsequently evaluated HAV, reporting >1 log_10_,  >2 log_10_, and >3.5 log_10_ TCID_50_ reductions after 10-min treatments of 300, 400, and 500 MPa respectively.

## Inactivation of HAV Within Foods

Subsequent studies were directed at characterizing the potential of foodborne viruses to be inactivated in foods such as oysters, green onions, and strawberry puree (highlighted in Table 1). Work with live oysters (*Crassostrea virginica*) contaminated with up to 6 log_10_ of HAV after exposure to HAV-contaminated seawater revealed that 350, 375, and 400-MPa treatments in a water-based Quintas QFP-6 (ABB Autoclave Systems, Inc., Columbus, OH) for 1-min at 9 °C inactivated >1, >2, and >3 log_10_ of HAV, respectively (Calci et al. 2005). Later comparison of HAV inactivation observed in shucked oyster meats typically used for research samples, and whole-in-shell oysters, as would be used commercially, confirmed similar inactivation (Kingsley et al. 2009). Inactivation of bioaccumulated HAV within Mediterranean (*Mytilus galloprovincialis*) and blue (*Mytilus edulis*) mussels was also performed. 5-min room temperature treatments of 350 and 400 MPa inactivated 1.7- & 2.9- and 2.1- & 3.6-log_10_ HAV within Mediterranean and blue mussels, respectively (Terio et al. 2010).

**Table 1:** High pressure processing performed on viruses associated with bivalve shellfish or produce

Virus	Matrix	Contamination method	Pressure tested	Temp/time tested	Amount inactivation	Citations
HuNoV	Shucked oyster	Injection	400-MPa	6 °C 5-min	Some protection of volunteers (estimate of 3-log inactivated)	Leon et al. (2011)
HuNov	Shucked oyster	Injection	400-MPa	22 °C 5-min	No protection of volunteers	Leon et al. (2011)
HuNoV	Shucked oyster	Injection	600-MPa	6 °C 5-min	4-log_10_ RT-PCR units	Leon et al. (2011)
MNV	Clams	Water uptake	300–500-MPa	10 °C 1-min	4-log_10_ at 500-MPa	Arcangeli et al. (2012)
MNV	Shucked oysters	Water uptake	300–400-MPa	5 °C 5-min	>4-log_10_ at 400-MPa	Kingsley et al. (2007)
MNV w/live mice	Oyster puree	Seeded	400-MPa	8 °C 5-min	Mice protected from 200 PFU	Gogal et al. (2011)
HAV	Shucked oysters	Water uptake	300–400-MPa	9 °C 3-min	>3-log_10_ at 400-MPa	Calci et al. (2005)
HAV	Shucked blue mussels	Water uptake	300–400-MPa	18–22 °C 5-min	3.6 log_10_ at 400-MPa	Terio et al. (2010)
HAV	Shucked med. mussels	Water uptake	300–400-MPa	18–22°C 5-min	2.9-log_10_ at 400-MPa	Terio et al. (2010)
HAV	Whole in-shell oysters	Water uptake	300–400-MPa	20 °C 5-min	2.6 log_10_ at 400-MPa	Kingsley et al. (2009)
HAV	Green onion slices	Overnight immersion	225–375-MPa	21 °C 5-min	4.75 log_10_ at 375-MPa	Kingsley et al. (2005)
HAV	Strawberry	Puree (mixed as 4 parts strawberry: 1part HAV stock	225–375-MPa	21 °C 5-min	4.32 log_10_ at 375-MPa	Kingsley et al. (2005)
MNV	Strawberry	Puree (mixed to 10^7^PFU/gram	350–450-MPa	4 °C 2-min	2.4 log_10_ At 350-MPa	Lou et al. (2011)

*HuNoV* human norovirus, *MNV* murine norovirus, *HAV* hepatitis A virus

For HPP treatment of HAV and strawberries (Kingsley et al. 2005), a puree was made and mixed with HAV in DMEM at 4 parts strawberry puree and 1 part HAV in cell culture media. For HAV and green onions, the onions were chopped into slices approximately 1 cm in size and soaked overnight with HAV stock in a rotating vessel. A 350 MPa, 5-min treatment at an initial temperature of 21 °C in a water-based Avure PT-1 high pressure unit (Avure Technologies, Inc., Kent, WA) was sufficient to inactivate 4 log_10_ of HAV within the context of either strawberry puree or sliced green onions (Kingsley et al. 2005). It was noted that, in the context of strawberry puree, HAV inactivation was more sensitive to HPP than virus stocks in DMEM tissue culture media. HAV has also been evaluated in mineral water and sausages. A 5-min, 500-MPa treatment at 4 °C gave a 3.29-log_10_ reduction and 1.1-log_10_ reduction in water and sausages, respectively (Sharma et al. 2008).

## Pressure Sensitivity of HuNoV Surrogates

Direct assessment of human norovirus viability requires the use of human volunteers. Therefore, initial work assessing the feasibility of inactivating HuNoV was performed using the surrogate viruses, feline calicivirus (FCV), and murine norovirus (MNV). Testing an FCV stock in DMEM culture media indicated that a 5-min, 275-MPa, room temperature treatment in a custom-built oil-based unit was sufficient to inactivate 7 log_10_ of this virus (Kingsley et al. 2002), suggesting potential for inactivation of HuNoV. Buckow et al. (2008) also did extensive work characterizing and modeling FCV inactivation by HPP.

A few years later, MNV was discovered (Wobus et al. 2006). Based on its genetic classification as a norovirus, its ability to infect mice orally, and to replicate in the murine gastrointestinal tract, it was generally judged a superior surrogate to FCV since FCV infects felines via a respiratory route and though classified as a calicivirus, it is not a member of the norovirus family. Initial evaluation of MNV stocks in DMEM tissue culture media showed that MNV was sensitive to pressure after 5-min treatments above 350 MPa at room temperature with a 5-min, 450-MPa treatment being sufficient to inactivate 6.85 log_10_ of MNV using the Avure PT-1 unit (Kingsley et al. 2007). Thus, MNV was found to be less sensitive to pressure than FCV.

## Inactivation of HuNoV Surrogates Within Foods

To investigate the feasibility of MNV inactivation within shellfish, live oysters were contaminated in a large flow-through oyster tank permitting simulated natural bioaccumulation of MNV to levels approximately 6 log_10_ per oyster. A 5-min, 400-MPa treatment at 5 °C was sufficient to inactivate 4 log_10_ of MNV (Kingsley et al. 2007). Later experiments were performed which showed that high pressure inactivation of MNV could be confirmed to an equal extent both by in vivo infection of mice and in vitro cell culture (Gogal et al. 2011). Inactivation of MNV within clams was recently demonstrated by Arcangeli and coworkers (2012). Kovač et al. (2012b) recently reported a 2.63-log_10_ reduction in strawberry puree after a 5-min, 300-MPa treatment and complete inactivation of MNV after a 400-MPa treatments of ≥1-min.

## HPP and Other Nonenveloped Viruses

To investigate other potential foodborne human picornaviruses, a number of other viruses were tested for pressure sensitivity (highlighted in Table 2). These viruses, coxsackie A9, coxsackie B5, polio, Aichivirus, and human parechoviruses, were suspended in tissue culture media (MEM or DMEM supplemented with fetal bovine sera). HPP inactivation was variable and in some cases very limited. Coxsackie B5, polio (Chat strain), and Aichivirus were completely resistant to 5-min, 600-MPa pressure treatments at room temperature using a custom-built oil-based pressure unit (Kingsley et al. 2004). Resistance of poliovirus to high pressure was observed previously (Wilkinson et al. 2001; Kingsley et al. 2002). For human parechovirus, a 5-min, 500-MPa treatment at room temperature resulted in a 4.3-log_10_ tissue culture infectious dose 50 % (TCID_50_) reduction (Kingsley et al. 2004). For coxsackie A9, a 5-min, 400-MPa treatment at room temperature resulted in a 3.4-log_10_ TCID_50_ reduction (Kingsley et al. 2004). In contrast, several non-foodborne picornaviruses, e.g., foot and mouth disease, bovine enterovirus, and rhinovirus have been shown to be quite sensitive to pressure (Goncalves et al. 2007; Murchie et al. 2007; Oliveira et al. 1999). Thus, it is clear that virus sensitivity to pressure is highly variable and cannot really be accurately predicted based on genetic classification. In fact, even different virus strains, as highlighted by coxsackie A9 and B5, can behave differently under pressure. A few other potential foodborne viruses, such as the rotavirus and adenovirus, which are common in human stool, as well as some bacteriophages, have been proposed as surrogates for nonenveloped pathogenic viruses and evaluated for HPP sensitivity. Rotavirus is relatively sensitive to HPP. Khadre and Yousef (2002) demonstrated an 8-log_10_ reduction after a 2-min, 300-MPa treatment at room temperature although a small proportion of the rotavirus was noted to be highly resistant to pressure. Four hundred MPa treatments appear to be sufficient to inactivate adenovirus type D and AdV2 (Kovač et al. 2012a; Wilkinson et al. 2001). Evaluation of HEV and sapovirus sensitivity to HPP is currently a research need. A number of phages have been evaluated for barosensitivity (Guan et al. 2006; 2007; Sheldon et al. 2008; Smiddy et al. 2006). As with animal viruses, the various bacteriophage types evaluated also display variable sensitivity to pressure.

**Table 2:** High pressure processing performed on HAV and other picornaviruses

Virus	>1 log Inactivation observed at	Substantial inactivation pressure (>3 log_10_)	Citation
HAV^a^	325-MPa	400-MPa	Kingsley et al. (2002; 2006)
Aichi^a^-	Resistant to 600-MPa	Resistant to 600-MPa	Kingsley et al. (2004)
Parecho^a^-	400-MPa	500-MPa	Kingsley et al. (2004)
Coxsackie A9^a^	n/d	400-MPa	Kingsley et al. (2004)
Coxsackie B5^a^	Resistant to 600-MPa	Resistant to 600-MPa	Kingsley et al. (2004)
Polio^a^	Resistant to 600-MPa	Resistant to 600-MPa	Kingsley et al. (2004)
FMDV^b,c^	n/d	240-MPa	Oliveira et al. (1999)

*n/d* not determined

^a^5-min treatment

^b^Urea was present in the pressure-treated sample

^c^Treament time was ~1 h

## Matrix and HPP Treatment Conditions

**Fig. 1:**
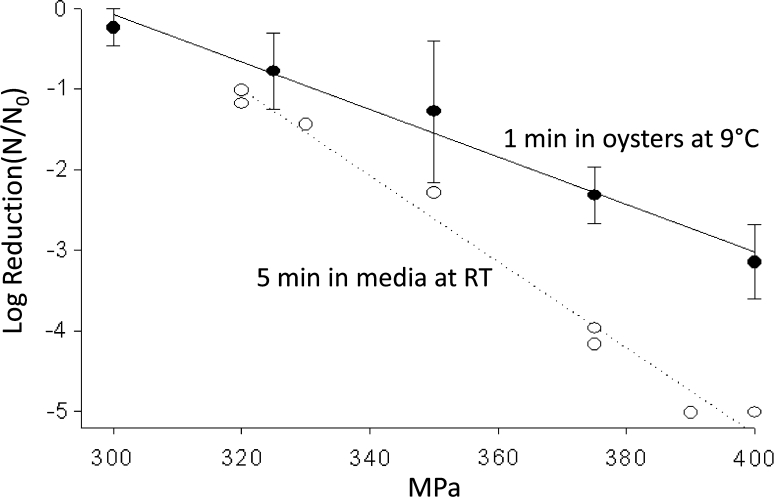
Hepatitis A virus sensitivity to pressure suspended in cell culture media and within oysters. *Open circles* denote individual 5-min treatments at room temperature in a custom-built oil-based unit. *Dark circles* denote average of three trials for 1-min HPP treatments within oysters performed separately in a Quintas QFP-6 at 9 °C. *Error bars* denote SE

**Fig. 2:**
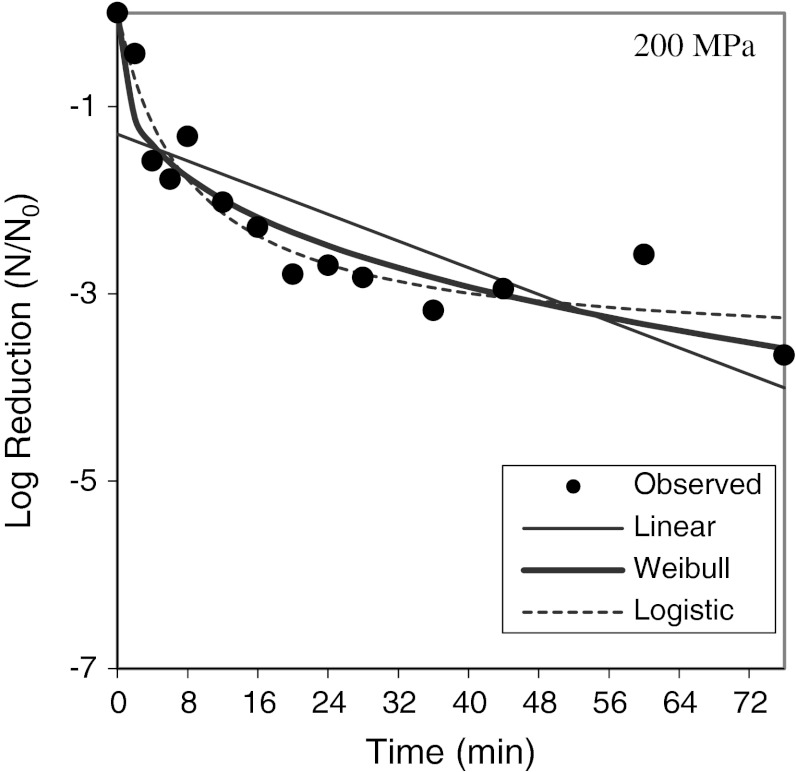
Effect of extended treatment time on feline calicivirus. *Dark circles* indicate the average of three trials. Results are modeled against Weibull and log-logistic curves

**Fig. 3:**
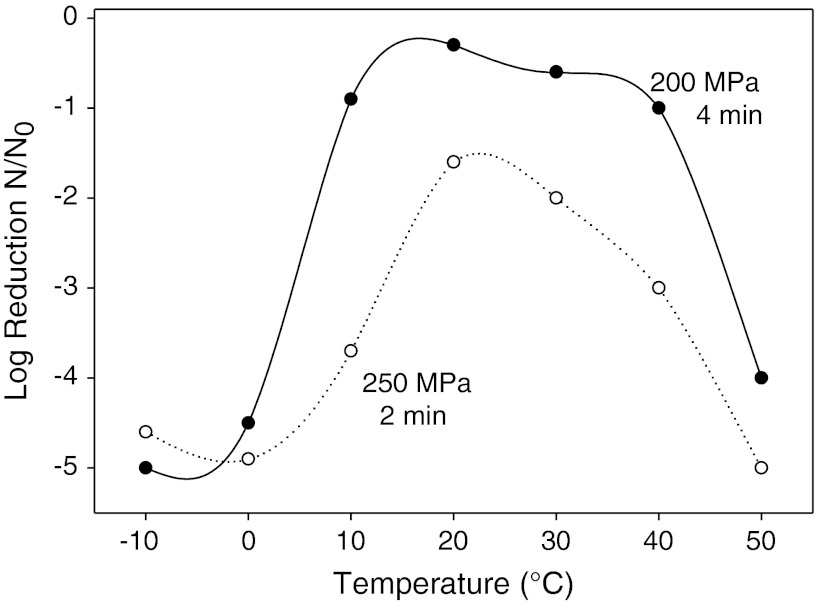
Effect of temperature on pressure inactivation of feline calicivirus. Average log reduction observed from three trials evaluating initial temperatures from −10 to +50 °C are shown for 4-min 200-MPa (*dark circles*) and 2-min 250-MPa treatments (*open circles*)

**Fig. 4:**
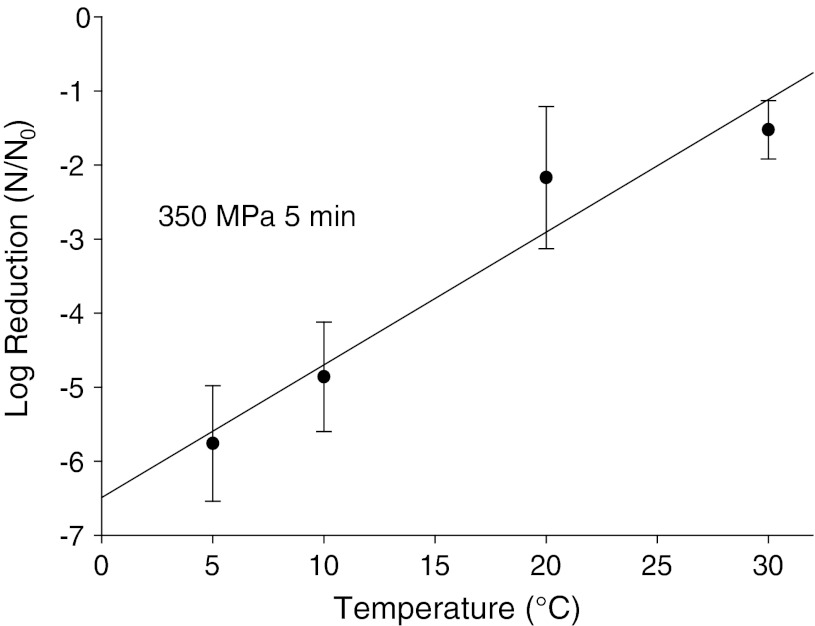
Effect of temperature on pressure inactivation of murine norovirus. Average log reduction observed for three trials applying 350 MPa for 5 min at initial temperatures ranging from 0 to 30 °C. *Error bars* denote SE

Beyond just testing viruses in individual food matrices for HPP sensitivity, it was important to focus on defining HPP parameters which influence the inactivation of viruses. Plotting the log of virus reduction versus pressure levels applied generally gives a straight line indicating a first-order relationship between applied pressure and inactivation observed (Fig. 1). For FCV, MNV, and HAV, extended treatment time at a given pressure resulted in increased inactivation, but that increase diminished with time. As shown in Fig. 2, modeling inactivation curves of pressure applied versus variable time applied reveals inactivation curves which most closely fit Weibull and log-logistic curves (Chen et al. 2005; Kingsley et al. 2006; 2007). Oscillatory high pressure processing for 2, 4, 6, and 8 cycles from 0 to 400 MPa did not considerably enhance pressure inactivation of HAV as compared with continuous high pressure (Kingsley et al. 2006). For the initial temperature at which pressure is applied, there were considerable differences in virus inactivation behavior. For the two caliciviruses tested, MNV and FCV, reduced temperatures resulted in dramatically enhanced inactivation (Figs. 3, 4). In fact, room temperature treatment in the water-based Avure PT-1 machine was the least effective temperature for FCV with 4-min, 200-MPa treatments at −10 and 50 °C resulting in 5.0 and 4.0-log_10_ reductions, respectively, while treatment at 20 °C resulted in only a 0.3-log_10_ reduction (Chen et al. 2005). For murine norovirus, 5-min, 350-MPa treatments at 20 and 30 °C resulted in 1.79- and 1.15-log_10_ reductions, respectively, while treatment at 5 °C resulted in a 5.56-log_10_ reduction (Kingsley et al. 2007). The idea that water has a density maximum at 4 °C suggested an appealing hypothesis that refrigeration temperature may be optimal for HPP inactivation (Dumay et al. 2006). However, confounding the hypothesis that cooler temperatures will generally enhance inactivation, HAV in DMEM tissue culture media was found to be enhanced by warmer temperatures and markedly reduced at colder temperatures. For example, 1-min treatments of 400-MPa at −10, 20, and 50 °C reduced HAV titers by 1.0-, 2.5-, and 4.7-log_10_ PFU/ml, respectively (Kingsley et al. 2006). Thus, it is clear that while temperature is a key consideration for HPP targeting viruses, the appropriate temperature will depend on the specific virus targeted.

Since HPP is increasingly applied to processed and acidic foods, it was desirable to evaluate the potential influence of some common food components, such as salt, sugar, and acidic pH, on the efficiency of HPP against viruses. Work with FCV showed that elevated NaCl and sucrose levels substantially reduce HPP inactivation of FCV (Kingsley and Chen 2008). For example a 5-min, 250-MPa treatment at 20 °C with no sucrose added to the virus stock in DMEM tissue culture media was reduced by 5.1 log_10_ pfu/ml. However, adjusting the FCV stock to 40 % sucrose resulted in only a 0.9 % reduction (Kingsley and Chen 2008). Addition of NaCl to a final concentration of 12 % reduced the efficiency of FCV inactivation from 5.1 log_10_ PFU/ml to only 0.7 log_10_ PFU/ml for a 5-min, 250-MPa treatment at 20 °C (Kingsley and Chen 2008). Work with HAV has also shown that NaCl also reduces inactivation in a similar fashion (Kingsley and Chen 2009). Presumably, this “solute effect” has something to do with preventing higher density packing of H_2_O around the solvation cage of the protein thereby reducing protein denaturation. Although it is clear that HPP is a water-dependent technology, its effect is not simply a function of water activity since it was noted that FCV samples with identical water activity and containing either enhanced amounts of NaCl or sucrose had differing amounts of inhibition (Kingsley and Chen 2008). Divalent cations (Ca^2+^ and Mg^2+^) are generally known to stabilize viruses and bacteriophages. Interestingly, Sanchez and coworkers (Sánchez et al. 2011) have shown that 10 mM CaCl_2_ was highly protective against HPP inactivation of MNV.

Lou et al. (2011) have shown that MNV is less sensitive to HPP under acidic conditions, and observed a reduced inactivation in acidic aqueous media, as well as in acidified strawberry and carrot purees. As noted earlier, results with HAV indicate that inactivation is enhanced when pressure is applied at lower pH (Kingsley and Chen 2009), contradicting the recent results observed for the HuNoV surrogate, MNV (Lou et al. 2011). Mechanistic explanations as to why HPP inactivation of HAV is enhanced, and MNV inactivation is reduced by H^+^, are currently elusive. Of general note when performing HPP, the behavior of weak acids and bases can be substantially altered under pressure since the ionic dissociated side of the chemical reaction is often favored under pressure because the dissociated ions occupy less space than nondissociated forms. Thus, for a weak acid like acetic acid, increasing pressure causes more disassociation (HOAc → H^+^ and OAc^−^) resulting in a lower pH under pressure (Kitamura and Itoh 1987).

## HPP and HuNoV

Given that HuNoV is now the most common foodborne etiologic agent and both MNV and FCV were found to be pressure sensitive, direct testing of HPP inactivation was highly desirable. Consequently, a human volunteer study involving HPP treatment of HuNoV-contaminated oysters was performed (Leon et al. 2011). This was accomplished by injecting 10^4^ RT-PCR units of GI.1 norovirus (Norwalk strain 8fIIb) into pressure-shucked oysters. A 5-min, 400-MPa treatment at 25 °C was not sufficient to inactivate the virus. Testing a second volunteer group with 5-min-, 600-MPa-treated virus at 6 °C indicated that the virus was completely inactivated. A third group was fed NoV-contaminated oysters after a 5-min, 400-MPa treatment at 6 °C. This treatment reduced the numbers of volunteers who became sick, but did not completely protect all volunteers. Thus pressures of at least 400-MPa or higher would be required to make human norovirus-contaminated shellfish safe for consumption. Based on the reduction of human volunteers, it was postulated that the 400-MPa, 6 °C treatment probably inactivates between 3- and 4 log_10_ of human norovirus (Leon et al. 2011; supplemental material). This conclusion is also supported by subsequent research which has shown a dramatic drop in HuNoV’s ability to bind to virus receptor-like swine mucin glycoproteins after a 5-min, 400-MPa treatment at 5 °C (Dancho et al. 2012). This drop was not observed for a 5-min, 300-MPa treatment at 5 °C (Dancho et al. 2012) which is not sufficient to inactivate HuNoV. The volunteer study also confirmed that colder temperatures did enhance the inactivation of human norovirus as was observed for the norovirus surrogates FCV and MNV since complete inactivation of HuNoV was observed when pressure was applied at 6 °C and not 25 °C for 400-MPa treatments. It is difficult to predict how HuNoV would behave in other foods; but, given that shellfish are high salt foods (2**–**3 %), it is conceivable that somewhat greater inactivation would be observed in lower ionic strength environments.

## HPP Inactivation Mechanism of Viruses

How HPP actually inactivates foodborne viruses has not been extensively delineated, but all indications are that high pressure is altering the virus capsid or protein coat surrounding the positive-stranded RNA. Enteric viruses are non-enveloped and, by definition, do not contain lipid envelopes. Therefore, HPP inactivation of foodborne virus, unlike foodborne bacteria, has no lipid-specific component. High pressure generally does not disrupt covalent bonds and it is understood that high pressure does not damage the primary structure of nucleic acids, such as the RNA encoded within these viruses. It stands to reason, therefore, that HPP inactivation must be a function of pressure’s effect on virus protein conformations. Viewed from a capsid function perspective, the virus must attach to its host cell receptor, penetrate the cell membrane, and then release the RNA into the cytosol of the cell. Once inside the cytosol, the virus RNA genomes of picornaviruses and caliciviruses are functional mRNAs that are sufficient to initiate transcription and subsequent virus replication (Racaniello 2001). Thus, high pressure must cause a protein-mediated effect that prevents virus attachment, penetration of the host cell, or uncoating once the virus has entered the cell. A number of publications have shed some light on how HPP might inactivate viruses. It was shown that after inactivation of HAV at 500 MPa, the capsid was still able to protect the RNA genome from degradation by RNAse (Kingsley et al. 2002), indicating that inactivated HAV still had a relatively intact capsid. For a couple of non-foodborne picornaviruses, foot and mouth disease virus (FMDV) and rhinovirus, there is evidence that a part of the virus capsid, Vp4, is released as a result of high pressure treatment (Oliveira et al. 1999, Goncalves et al. 2007). Tang et al. (2010) has shown that 400-MPa**-**treated MNV is rendered defective for binding to its host cell, while subsequent evaluation by Lou et al. (2011) has demonstrated that 600 MPa is sufficient to destroy the integrity of the MNV capsid. As described earlier, different picornavirus have varying sensitivities to pressure. The reason for this difference has not been determined but one hypothesis is that it relates to the receptor-binding mechanisms (Kingsley et al. 2004). Human parechovirus and coxsackie A9 virus, two viruses that are sensitive to pressure, are known to interact with their host cell via a prominent peptide loop encoding an integrin-like RGD motif which protrudes from the capsid (Boonyakiat et al. 2001; Chang et al. 1989; Hughes et al. 1995). Polio and coxsackie B5, two other viruses that were resistant to 600-MPa treatments, are known to lack the RGD motifs and have canyon-like pits on the capsid surface that mediate receptor interactions (Bergleson et al. 1997; Racaniello 2001). It is conceivable that protruding receptor-binding domains might be more susceptible to pressure-mediated protein denaturation than canyon-like pits.

## Caveats and Contradictions

While a number of universal themes for HPP virus inactivation have emerged, such as first-order inactivation versus pressure and log-logistic inactivation curves for constant pressure application versus time of pressure application, there are also a number of contradictions. Clearly, temperature and pH are critical considerations that appear contradictory for HPP inactivation of HuNoV and HAV. For the three caliciviruses tested to date (MNV, FCV, and GI.1 HuNoV) all had enhanced inactivation at refrigeration temperature. Whether this pattern will prove valid for all caliciviruses remains to be determined. The extent to which elevated temperatures influence are beneficial for the inactivation of other picornaviruses, besides HAV, is also presently unknown. The issue of surrogate and strain differences is also a concern. It is clear that different but taxonomically related viruses can behave differently under pressure as exhibited by the difference between FCV and MNV and the range of pressure sensitivities observed for the picornaviruses. In the case of HPP effectiveness, assumptions that a surrogate will behave in a manner analogous to the human pathogenic viruses may not be valid. Furthermore, the potential of different strains of the same virus type to have differing sensitivity to pressure, as exhibited by coxsackie A9 and B5, is a concern. Presently, the degree to which HuNoVs and wild-type HAV strains may vary in pressure sensitivities is an open question. The HuNoV HPP volunteer study evaluated only one HuNoV strain. Noroviruses are genetically highly diverse and it is not currently known whether this would translate into a diverse sensitivity range for these viruses. Most HPP inactivation research has also been with one strain of tissue culture-adapted HAV, which is presumably no longer pathogenic. Shimasaki et al. (2009) did evaluate different strains of HAV for HPP sensitivity and did identify one strain which was more sensitive to HPP. However, the other three strains had comparable sensitivity to the tissue culture-adapted HM-175 strain described here. Another important caveat is that since HPP targets the capsid and not the virus genome, RT-PCR- and PCR-based protocols will most likely still detect the presence of the viruses in foods even though these viruses may have been inactivated.

In conclusion, both HuNoV and HAV can be inactivated by HPP. Research described here can serve as a preliminary guide to whether a current commercial process could be effective against HuNoV or HAV. However, given the complexities of food matrices and variable response of different viruses, direct validation of HPP conditions within the food or food matrix being produced will be required. Considerations about food product formulations and subsequent alterations to those formulations will need to account for water activity and the effects of ionic strength, dissolved sugar levels, and other solutes. It is important to recognize that specific strategies which enhance the inactivation of HAV may inhibit inactivation of HuNoV (i.e., temperature and acidity) and vice versa. It is evident that current commercial HPP protocols performed at ambient water temperatures for a few minutes at 275 or 300 MPa to shuck oysters and as a *Vibrio* intervention likely would not have a substantial effect on HAV or HuNoV. However, for shellfish destined to be cooked, it is conceivable that higher pressure application or 400 or 500 MPa could be applied to sanitize shellfish without discernible organoleptic changes after cooking. At present, a universal HPP strategy to inactivate all potential foodborne viruses in a given food under conditions that would not significantly alter organoleptic food qualities still presents a substantial challenge.
